# Membrane Fusion-Based Mirabilis Himalaica-Derived Exosome-like Nanoparticles Fused with Cell-Penetrating Peptide Mediated for Chebulinic Acid Delivery Against UVA-Induced Photoaging

**DOI:** 10.3390/cells15141235

**Published:** 2026-07-08

**Authors:** Weiwei Zhao, Siqi Yang, Ruobing Liu, Chaozhi Liu, Jing Zhang, Ying Liu, Guihong Sun, Mingxiong Guo

**Affiliations:** 1 Key Laboratory of Biodiversity and Environment on the Qinghai-Tibet Plateau, Ministry of Education, School of Ecology and Environment, Xizang University, Lhasa 850000, China; 2 Hubei Key Laboratory of Cell Homeostasis, College of Life Sciences, Wuhan University, Wuhan 430072, China; 3 Hubei Provincial Key Laboratory of Allergy and Immunology, Taikang Medical School (School of Basic Medical Sciences), Wuhan University, Wuhan 430071, China

**Keywords:** anti photoaging, chebulinic acid, plant-derived exosome-like nanoparticles, TAT peptide

## Abstract

**Highlights:**

**What are the main findings?**

- Chebulinic acid (CA), a bioactive polyphenol from Terminalia chebula, is confirmed to mitigate UVA-induced skin photoaging, expanding its anti-UVR damage application from UVB to UVA exposure.
- A membrane hybridization strategy is developed to repurpose non-PELN large particles (>1000 nm) into TAT-anchored extruded MELNs (eMELNs, ~200 nm), which effectively delivers CA across the skin barrier.

**What are the implications of the main findings?**

- The TAT-anchored eMELN nanocarrier provides a green, biosafe and efficient transdermal delivery strategy by utilizing “waste” particles generated during PELN preparation, improving biological resource utilization.
- This study fills the gap in natural product-based UVA protection strategies, laying a foundation for developing next-generation natural anti-photoaging agents and promoting translational application of CA and PELNs in dermatology.

**Abstract:**

Exposure to ultraviolet (UV), particularly UVA radiation, is a primary driver of photoaging due to its deep dermal penetration, which triggers DNA damage, collagen degradation, and immune suppression. Chebulinic acid (CA), a polyphenolic compound from *Terminalia chebula*, exhibits potent antioxidant and anti-inflammatory properties against UVB-induced skin damage. However, its large molecular weight hinders transdermal delivery and the TAT_47–57_ peptide (core of HIV-1 TAT) enables rapid transmembrane transport. Large particles with double-layer membrane structure and a diameter exceeding 1000 nm were obtained during the separation of plant-derived exosome-like nanoparticles (PELNs), which are not considered as PELNs (50–500 nm), after a mixture with TAT anchored to the surface of engineered artificial vesicles (EAVs) and extrusion causes membrane fusion, employed as novel nanocarriers to overcome the difficulty in skin penetration by leveraging their lipid bilayer structure and surface membrane-anchored TAT for efficient epidermal fusion and intercellular penetration. Furthermore, CA-loaded TAT-ePELNs demonstrate significant efficacy in mitigating UVA-induced photoaging. Collectively, this study expands the anti-UVR damage application spectrum of CA from UVB to UVA exposure and establishes a green, efficient, and biosafe strategy for transdermal drug delivery by utilization of non-PELNs generated during the preparation process of PELNs.

## 1. Introduction

Chronic repetitive ultraviolet irradiation exerts profound biological impacts on skin tissue, triggering DNA mutagenesis, cellular apoptosis, dermal structural remodeling, inflammatory cascades and dysregulated immune activity. Collectively, these pathological alterations drive clinical skin disorders including hyperpigmentation, cutaneous inflammation, photoimmunosuppression, solar urticaria, photoaging and photocarcinogenesis [1]. UVA rays constitute more than 90% of the solar ultraviolet radiation reaching the Earth’s surface and act as the primary inducer of photoaging. Among all ultraviolet wavelengths, UVA possesses the greatest skin penetration capacity, enabling it to permeate deep dermal layers [2,3]. This makes UVA-induced photoaging a critical clinical challenge, particularly in high-altitude regions like Qinghai-Xizang Plateau—where extreme environmental conditions (cold, hypoxia, intense UV radiation, prolonged daylight, etc.) [4] exacerbate skin damage risks.

Natural Chinese medicinal products offer promising solutions for UVR protection, and *Terminalia chebula*—hailed as the “King of Tibetan Medicine” [5]—stands out as a potential candidate. Our prior research confirmed that chebulinic acid (CA), a major bioactive polyphenol in *T. chebula*, effectively mitigates UVB-induced skin damage by scavenging reactive oxygen species (ROS) and exerting anti-inflammatory, antiapoptotic effects [6]. Given ROS are a common mediator of both UVA- and UVB-induced skin injury [3,7], we hypothesized CA may also counter UVA-induced photoaging—a critical knowledge gap, as UVA’s dominant role in photoaging remains underaddressed by natural product-based interventions.

However, the therapeutic potential of CA is constrained by the formidable barrier of the skin due to its molecular weight (~956 Da) exceeding the 500 Da threshold for passive transdermal diffusion [8] and plant-derived exosome-like nanoparticles (PELNs) have been employed as transdermal delivery nanocarriers of CA in our previous study. PELNs possess several advantages: (1) PELNs’ abundant production compares with mammalian cell-derived exosomes (MDEs); (2) their plant sources can be easily and economically grown and obtained, unlike human cell cultures; (3) their lipid bilayer structure (50–500 nm in diameter) facilitates fusion with epidermal membranes and high deformability allows for intercellular penetration without compromising the integrity of the skin barrier; (4) their superior biodegradability and biocompatibility compares with synthetic nanocarriers [9,10,11,12]. PELNs exert combinatorial therapeutic benefits against multiple dermatological disorders, including photoaging, hair loss, hyperpigmentation and cutaneous wound defects. Such protective outcomes are achieved through diverse biological pathways: neutralizing reactive ROS, tuning inflammatory signaling cascades, and governing the production of inflammatory cytokines [13,14,15].

To address both the CA delivery barrier and resource waste issue, we developed a suite of innovative strategies. First, using the method developed in our previous study [16], we leveraged TRP-PK1 (a synthetic peptide that conjugates to other peptides and integrates into cell membranes) [16,17] to anchor TAT, a protein transduction domain, with an 11-amino-acid core (TAT_47–57_), enabling rapid transmembrane cargo transport [18,19,20,21,22] to engineered artificial vesicles (EAVs). This non-covalent modification enhances EAV penetration without the cytotoxicity of chemical crosslinkers, breaking from conventional covalent surface modification protocols. More groundbreakingly, we pioneered a membrane hybridization strategy to repurpose the large “waste” particles. Many large particles with double-layer membrane structure (>1000 nm) were obtained in the sediment at a centrifugal speed of 18,000 *g*; they are not considered as PELNs (50–500 nm) [23] and are rarely studied, and we named the sediment *Mirabilis himalaica* PELNs (MELNs) p18. Nanoparticle size is an important factor for transdermal penetration and particles larger than 500 nm may be difficult for the skin to absorb [24]. We efficiently utilize these large particles by mixing HaCaT cell-derived EAVs with MELNs p18 and extruding them through a filter membrane for membrane hybridization to obtain extruded MELNs (eMELNs) with TAT peptide (~200 nm).

**Scheme 1:**
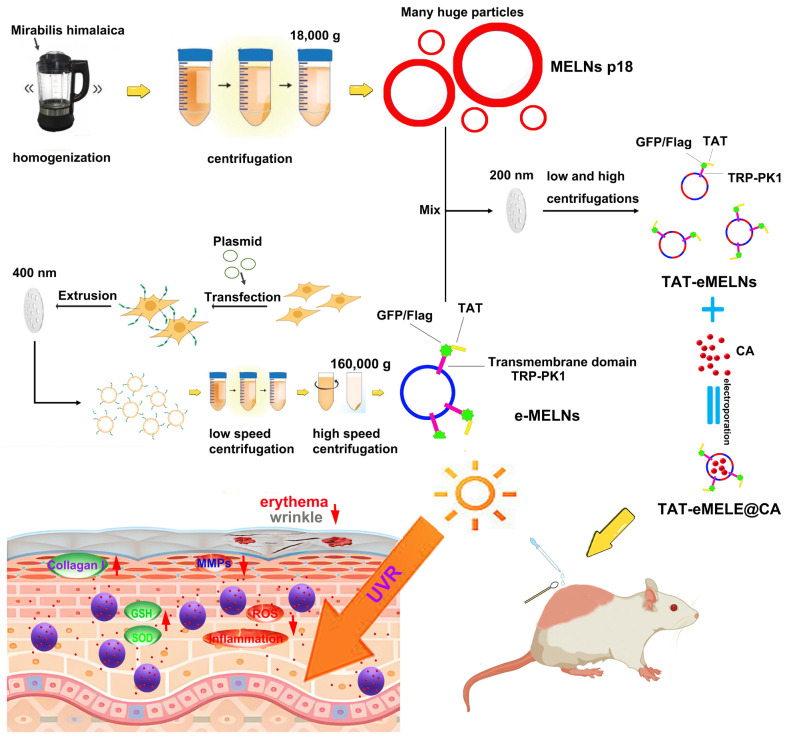
Schematic diagram of the formation and application of TAT-eMELNs.

This strategy firstly avoids the additional chemicals to mitigate apprehensions regarding biosafety risks, then we transform non-PELN large particles into valuable carriers, markedly improving the utilization rate of biological resources. Lastly, the membrane surface anchoring of TAT dramatically enhances the cellular and transdermal absorption efficiency of EAVs. Moreover, we load CA, which can effectively resist skin damage caused by UVB, into eMELNs with TAT peptide through electroporation, and validate the anti-UVA function of CA and TAT-eMLNs@CA in vivo and in vitro (Scheme 1). Building on our prior work [6], this study expands the translational potential of CA and PELNs in dermatological therapeutics and fills a critical gap in UVR protection strategies by targeting UVA-induced photoaging, laying the foundation for the development of next-generation, natural product-based anti-photoaging agents.

## 2. Materials and Methods

### 2.1. Experimental Materials

Chebulinic acid (98.46%) was purchased from Chengdu Pufei De Biotech Co., Ltd. (Chengdu, China). The chebula fruit (*Terminalia chebula* Retz.) used in this study was produced in Nepal, and the production area of *Mirabilis himalaica* is Linzhi City, China. A bicinchoninic acid protein assay kit was purchased from KeRui (Wuhan, China). DiI was supplied by Beyotime (Shanghai, China). Antibodies against CD63 (25682-1-AP), TSG101 (28283-1-AP), MMP1 (83114-2-RR), MMP3 (82984-4-RR) were purchased from Proteintech (Wuhan, China), and LaminB1 (MABT831) was purchased from Sigma-Aldrich (Sigma, St. Louis, MO, USA), and GAPDH (AC001) was purchased from ABclonal (Wuhan, China). CCK-8 was purchased from TopScience (Shanghai, China). Annexin V-FITC, EdU and cell senescence detection kits were purchased from Beyotime (Shanghai, China). HaCaT and HFF cell lines were obtained from the Cell Bank of the Chinese Academy of Sciences (Shanghai, China) and maintained in Dulbecco’s modified Eagle’s medium (DMEM; Monad, Wuhan, China). Culture medium for all cells was supplemented with 10% fetal bovine serum (VivaCell, Shanghai, China), 100 U/mL penicillin and 100 U/mL streptomycin (Bio-Channel, Nanjing, China); incubation was performed at 37 °C under an atmosphere of 5% CO_2_.

### 2.2. Isolation and Purification of the ePELNs and EAVs

Fresh plant tissues were homogenized using a high-speed tissue grinder, and the resulting liquid filtrate was collected to eliminate bulky solid residues and impurities. The crude mixture underwent sequential low-speed centrifugation: first at 300× *g* for 10 min, then at 2000× *g* for another 10 min to clear coarse debris. The resultant supernatant was transferred into new sterile centrifuge tubes and subjected to 18,000× *g* centrifugation for 30 min to harvest the p18 precipitate. The pelleted nanoparticles at the tube bottom were resuspended in PBS buffer for subsequent collection.

Cells transfected with target plasmid DNA were digested with trypsin, harvested via centrifugation, rinsed twice with PBS, and resuspended in fresh PBS to prepare homogeneous cell suspension. The suspension was extruded through a liposome extruder to generate crude EAVs, which were further purified via stepwise ultracentrifugation. The extruded EAV suspension was sequentially spun at 300× *g* (10 min) and 2000× *g* (10 min) to remove coarse debris, followed by 18,000× *g* centrifugation for 30 min to discard large vesicle contaminants; a final 90 min centrifugation at 160,000× *g* was performed to pellet purified EAVs.

Pre-obtained MELNs p18 and EAVs were fully mixed and co-extruded using the liposome extruder. The mixed suspension was purified through a series of differential centrifugation steps: 300× *g* for 10 min and 2000× *g* for 10 min to remove coarse debris, 18,000× *g* for 30 min to deplete oversized vesicles, and a final spin at 160,000× *g* lasting 90 min to precipitate the target eMELNs.

### 2.3. Characterization of the Nanoparticles

A Malvern particle size analyzer (Zetasizer Nano ZS90, Malvern Panalytical, Great Malvern, UK) was utilized to characterize the particle size distribution and zeta potential of PELNs. Transmission electron microscopy (TEM, HT7700, Hitachi, Tokyo, Japan) was adopted to visualize the morphological features of these nanoscale vesicles. Briefly, nanoparticle samples were loaded onto copper grids and incubated for 5 min, followed by negative staining with 1% uranyl nitrate solution over a 30 s period. After air-drying, the prepared grids were imaged under TEM with an accelerating voltage set to 80 kV. Total protein content was extracted from the PELN samples and quantified spectrophotometrically using a BCA protein assay kit (MA0082, MeilunBio, Dalian, China).

### 2.4. Therapeutic Cargo Loading into Nanoparticles

CA and nanovesicle samples were softly blended within 100 μL electroporation EL transfection buffer under 4 °C conditions. Electroporation was then conducted at 180 V, with a 100 μs pulse width and a 1000 ms pulse interval, after which the resulting mixture underwent incubation at 37 °C. Unloaded CA used as a surfactant is not removed [6]. PBS was supplemented to the CA-loaded nanoparticle suspension to reach a final total volume of 1 mL. A fluorescence spectrophotometer (SynergyH1, BioTek, Winooski, VT, USA) was adopted to record the fluorescence intensity at a wavelength of 280 nm. The CA encapsulation efficiency and drug loading capacity were computed relying on the standard calibration curve of CA dissolved in PBS.

### 2.5. Removal of Free CA and Calculation of Encapsulation Efficiency

After electroporation of CA into the eMELNs, the sample was immediately subjected to ultracentrifugation at 160,000× *g* for 90 min at 4 °C. Following centrifugation, the CA-loaded eEMLNs were pelleted at the bottom, while the unencapsulated free CA remained in the supernatant. The supernatant was carefully aspirated, and the pellet was washed once with PBS to ensure complete removal of free CA.

To determine the encapsulation efficiency, an equal volume of the same batch of electroporated sample (containing both encapsulated and free CA) was collected before the removal step and lysed with an equal volume of 0.5% Triton X-100 (or 1% SDS) to release the total CA. This lysate was designated as the “total CA fraction.” Meanwhile, the supernatant collected after ultracentrifugation (containing free CA) was filtered through a 0.22 μm membrane and designated as the “free CA (non-encapsulated fraction)”.

The concentrations of CA in both fractions were quantified by UV-Vis spectrophotometry. The encapsulation efficiency was calculated as: EE% = [(C_total − C_free)/C_total] × 100%.

C_total is the CA concentration in the Triton X-100 lysate (total CA), and C_free is the CA concentration in the supernatant (free CA). Using this method, we obtained an encapsulation efficiency of approximately 55%, corresponding to ~45% of the initial CA remaining in the free form.

### 2.6. Plasmid Amplification and Extraction

Competent cells were thawed on ice and mixed with the target plasmid. After incubation on ice for 30 min, the mixture was heat-shocked at 42 °C for 90 s and immediately cooled on ice for 3 min. LB liquid medium was added, and the cells were incubated at 37 °C with shaking for 60 min. Bacterial suspension was centrifuged at 3000× *g* for 3 min, and the pellet was resuspended and spread on antibiotic-resistant agar plates for overnight culture at 37 °C.

Single colonies were inoculated and cultured in liquid medium. Bacteria were harvested by centrifugation at 3000× *g* for 10 min, and plasmids were extracted using a commercial plasmid purification kit according to the standard protocol. Briefly, bacterial pellets were resuspended in Solution I, lysed with Solution II, and neutralized with pre-cooled N3 buffer. After high-speed centrifugation at 12,000× *g* at 4 °C, the supernatant was treated with ETR solution and absolute ethanol for purification. The processed samples were loaded onto NA adsorption columns, washed sequentially with GPS, HBC, and Washing buffers, and finally eluted with Elution buffer. The concentration and purity of the extracted plasmid DNA were determined using a Nanodrop spectrophotometer.

### 2.7. Cell Transfection

Cell transfection was performed using Lipofectamine 2000 reagent (Invitrogen, Carlsbad, CA, USA) following the manufacturer’s standard protocols, with all operations conducted using RNase-free pipette tips and EP tubes. All culture media were prewarmed to 37 °C before transfection. Cells were cultured in 6-well or 12-well plates until reaching approximately 50% confluence, and reagent dosages were halved for 12-well plates compared with 6-well plates.

For 6-well plate transfection, 2000 ng of plasmid DNA or 100 pmol of siRNA (including negative control sequences) was diluted in 250 μL of serum-free medium. Meanwhile, 5 μL of Lipofectamine 2000 reagent was diluted in another 250 μL of serum-free medium and incubated for 5 min at room temperature. The two dilutions were gently mixed and incubated for 20 min to form lipid–nucleic acid complexes.

After removing the original culture medium, cells were supplemented with 1.5 mL of serum-free medium to facilitate cellular uptake of exogenous substances. Subsequently, 0.5 mL of the prepared transfection complex mixture was added to each well and mixed gently. After 6 h of incubation, the serum-free transfection medium was replaced with complete growth medium. Cells were harvested 48 h post-transfection, and transfection efficiency was verified by Western blot analysis. The dosages of plasmids and siRNA were adjusted appropriately according to the transfection efficiency for subsequent functional experiments.

### 2.8. ELISA Assay

All ELISA strips were equilibrated to room temperature before use. Standard wells were loaded with 50 μL of serially diluted standard solutions. For sample wells, 10 μL of each sample and 40 μL of sample diluent were added, while blank wells remained untreated. Each well (excluding the blank) was supplemented with 100 μL of HRP-conjugated detection antibody, followed by incubation at 37 °C for 60 min. After thorough washing five times with washing buffer, equal volumes of substrate A and B were added to each well and incubated at 37 °C for 15 min in the dark. The reaction was terminated by stop buffer, and the optical density (OD) values at 450 nm were measured within 15 min. A standard curve was established based on the OD values of gradient standards, and the target protein concentrations in samples were calculated using the linear regression equation.

### 2.9. In Vitro Cytotoxicity Assay

Cells were plated in 6-well or 96-well culture plates at densities of 50,000 cells per well and 5000 cells per well, respectively, followed by 24 h of incubation. Afterwards, cells were co-incubated with nanoparticles of varying concentrations and subsequently subjected to UVA radiation exposure.

CCK-8 assay (96-well format): Following the 24 h treatment period, 10 μL CCK-8 reagent was dispensed into every well and incubated for 1–3 h to facilitate quantitative detection. Optical absorbance at 450 nm was determined using a fluorescence spectrophotometer (SynergyH1).

EdU assay (6-well format): After 24 h of treatment, EdU working solution (C0071S, Beyotime) was supplemented to each well to reach a final concentration of 10 μM, with a 2 h incubation period. Cells were rinsed and fixed using 4% paraformaldehyde, then 500 μL click reaction mixture was added to each well and incubated in darkness at room temperature for 30 min. The proportion of proliferative cells displaying green fluorescence was counted under a fluorescence microscope.

β-galactosidase senescence staining (6-well format): Upon completion of the 24 h treatment, cells were rinsed and fixed with β-galactosidase staining fixative. A staining mixture consisting of 5 μL β-galactosidase staining solution A, 5 μL β-galactosidase staining solution B, 465 μL β-galactosidase staining solution C and 25 μL X-Gal solution (C0602, Beyotime) was then applied. The samples were incubated in the dark at room temperature for no less than 12 h, and the percentage of senescent cells stained green was quantified under a light microscope.

Intracellular ROS detection assay (6-well format): DCFH-DA (50101ES01, Yeasen, Shanghai, China) was diluted to a final concentration of 10 μM in each well, and cells were incubated with this probe at 37 °C for 30 min. After thorough washing, cellular ROS levels were analyzed by flow cytometry.

### 2.10. In Vitro Permeation Assay

Franz diffusion cells (FDCs; Taixinghukeboli, Taixing, China) were used for ex vivo permeation studies of the nanoparticle. Mouse skin was sandwiched between the donor and receptor compartments, with the epidermis facing the donor side. Approximately 0.5 mL of nanoparticle was loaded in the donor compartment, and 0.1 mL samples were withdrawn at specific time points from the receiver and quantified using a bicinchoninic acid protein detection kit.

### 2.11. In Vitro Transwell™ Model

Transwell™ chambers were adopted to build an in vitro simulated skin model. Identical quantities of HaCaT cells were seeded onto each upper insert well with 500 μL DMEM containing 10% fetal bovine serum. The lower compartment was filled with 1500 μL identical culture medium to balance osmotic pressure across the separating membrane. An equal number of HFFs were plated on the base of the lower chamber. Once cells reached full confluence within the chambers, DiI stained nanoparticles were introduced into the upper inserts and incubated for 48 h. Ultimately, cellular internalization of DiI-labelled nanoparticles was quantified and visualized with confocal laser scanning microscopy (Olympus, Tokyo, Japan) and flow cytometry, respectively.

### 2.12. Animal Experiments

All animal experimental protocols of the present research received ethical authorization from the Medical Ethics Review Committee of Xizang University and complied with the National Institutes of Health Guide for the Care and Use of Laboratory Animals. The dorsal hair of 8-week-old BALB/c mice was removed to expose a skin region measuring roughly 3 cm × 3 cm. Mice assigned to the UVA radiation group received daily UVA irradiation at a dosage of 2 J/cm^2^ over a continuous 8-week period. Meanwhile, 500 μL of PBS, free CA, F-eMELNs@CA or TF-eMELNs@CA was topically administrated to the irradiated skin site of each mouse every day prior to daily UVA exposure. Following intraperitoneal anesthesia with pentobarbitone, in vivo fluorescent signals emitted by DiI-labeled nanoparticles were captured using a small-animal imaging system (IVIS^®^ Lumina XRMS Series III, PerkinElmer, Waltham, MA, USA). Masson trichrome staining and TUNEL staining were performed to examine pathological alterations within cutaneous tissue.

### 2.13. Western Blot Analysis

Total cellular protein was isolated and quantified with a bicinchoninic acid protein detection kit. The harvested protein sample was mixed with 5× sodium dodecyl sulfate loading buffer for thermal denaturation at 100 °C over 10 min. Subsequently, 20 μg protein aliquots were resolved via 10% sodium dodecyl sulfate-polyacrylamide gel electrophoresis, electroblotted onto a nitrocellulose membrane, and sealed with 5% non-fat milk blocking solution for 1 h at room temperature. After three sequential rinses in Tris Buffered Saline with Tween (TBST), the membrane was incubated with horseradish peroxidase-labeled secondary antibody for another 1 h at room temperature. Target protein bands were imaged on the GeneGnome 5 imaging system (Syngene, Cambridge, UK).

### 2.14. Section Staining

The hearts, lungs, livers, spleens, and kidneys were dissected from each mouse, fixed with 4% paraformaldehyde and paraffin-embedded for HE staining by Servicebio (Wuhan, China). The back skins were peeled off from each mouse, fixed with absolute ethanol and paraffin-embedded for Masson or Tunel staining by Baiqiandu Bio (Wuhan, China).

### 2.15. Transcriptome Sequencing and Bioinformatics Analysis Methods

For transcriptome sequencing, all cellular samples were strictly processed under RNase-free and sterile conditions to ensure high-quality and non-degraded total RNA. Briefly, residual impurities and invalid components were rapidly removed from collected samples, and adequate Trizol reagent was immediately added to fully lyse cells and inhibit endogenous RNase activity, thereby locking the original gene expression profiles. After thorough homogenization, samples were snap-frozen in liquid nitrogen and stored at −80 °C without repeated freeze–thaw cycles. Qualified samples were subsequently delivered to a professional sequencing company for total RNA extraction, library construction, and high-throughput transcriptome sequencing.

After gene expression quantification and differential expression analysis, functional enrichment analysis was performed to systematically interpret the biological functions and signaling mechanisms of differentially expressed genes (DEGs). Gene Ontology (GO) enrichment analysis was conducted to annotate DEGs from three standardized dimensions: Biological Process (BP), Cellular Component (CC), and Molecular Function (MF). Meanwhile, Kyoto Encyclopedia of Genes and Genomes (KEGG) pathway enrichment analysis was applied to elucidate the systematic signaling networks and metabolic pathways involved in the DEGs.

All enrichment analyses were performed using a hypergeometric distribution test with all annotated genes as the background gene set. False discovery rate (FDR) correction was applied to eliminate false-positive results. GO terms and KEGG pathways with *p* < 0.05 or FDR < 0.05 were defined as significantly enriched.

### 2.16. Statistical Analysis

All statistical analyses were conducted using GraphPad Prism version 8.0.2 (GraphPad Software, San Diego, CA, USA). The majority of experimental data were normalized, and all results are expressed as the mean ± SD derived from three independent replicate experiments. Statistical differences between two independent groups were evaluated via Student’s *t*-test, while one-way analysis of variance (ANOVA) was applied for comparisons among three or more groups. A *p*-value of less than 0.05 was defined as the threshold for statistical significance.

## 3. Results

### 3.1. Preparation and Characterization of eMELNs

Previous studies have shown that Mulberry PELNs possess the strongest penetrability among seven PELNs in a simulated skin model; we further discovered that the penetrability of Mirabilis himalaica PELNs was stronger than the penetrability of Mulberry PELNs (Figure S1A). Concurrent studies conducted in our laboratory demonstrated that extruded Mirabilis himalaica PELNs exhibit robust ROS-scavenging activity, and the extrusion process does not cause significant alterations to their encapsulated bioactive components. Therefore, we selected Mirabilis himalaica as the raw material for PELNs. The method for isolations and purifications of the MELNs is as described in previous studies; many large particles with double-layer membrane structure and diameters in the nanometer range mixed with MELNs were found in the sediment (Figure S1B,D), obtained at a centrifugal speed of 18,000 *g*, and we named the sediment MELNs p18. MELNs p18 can be further extruded through a 200 nm filter membrane to obtain extruded MELNs (eMELNs). The yield of the p18 fraction was approximately 15 times higher than that of the classical PELNs obtained by ultracentrifugation. Given that the p18 fraction is normally discarded as a non-vesicular contaminant in conventional protocols, its substantially higher abundance makes it a more practical and scalable source for producing eMELNs.

The method for obtaining cell-derived EAVs is as described in previous studies, and Franz diffusion cells (FDCs) were used for ex vivo permeation studies. Due to biocompatibility reasons, the penetration of HaCaT cell-derived engineered artificial vesicles (EAVs) into mouse skin was approximately 2.5 times that of 293T cell-derived EAVs (Figure S1C), so we used HaCaT cell-derived EAVs instead. HaCaT cell-derived EAVs with different peptide segments were obtained by transfecting different plasmids into HaCaT cells extruded through a 400 nm filter membrane (Figure S1D). The plasmids encoded peptides with specific functional tags: TAT for enhanced cell penetration, GFP for fluorescence tracking, and Flag for immunodetection via Western blot. Then HaCaT cell-derived EAVs were mixed with MELNs p18 and extruded through a 200 nm filter membrane for crushing and fusing the membranes to obtain eMELNs with different peptide segments.

To determine the appropriate concentration ratio, we stained MELNs p18 with DiI before mixing with TAT-GFP-HaCaT cell-derived EAVs to obtain DiI stained TAT-GFP-eMELNs so that red fluorescence only derives from MELNs p18 and green fluorescence only derives from TAT-GFP-HaCaT cell-derived EAVs, and incubated HaCaT cells with DiI stained TAT-GFP-eMELNs (concentrations are all 100 μg/mL) to observed changes in fluorescence intensity in the cells. It could be observed that the green fluorescence significantly increased with the proportion of TAT-GFP-EAVs increase in the raw material. It is noteworthy that the red fluorescence still significantly increased with a decrease in the proportion of DiI stained eMELNs, indicating that the penetration of DiI stained TAT-GFP-eMELNs was gradually enhanced due to carrying more TAT peptides from TAT-GFP-EAVs (Figure S1E).

### 3.2. Characterization and Validation of eMELNs

**Figure 1:**
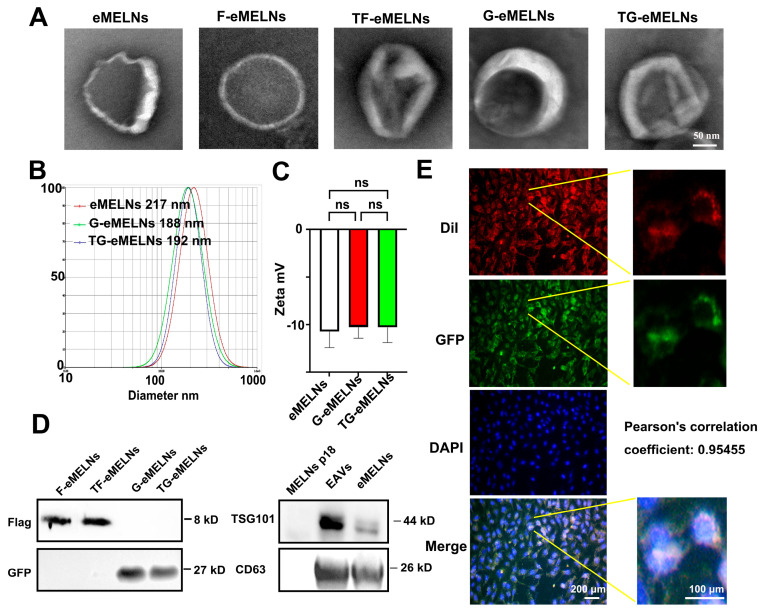
Morphology and verification of eMELNs. (**A**) Morphology of eMELNs characterized using TEM. Scale bar: 50 nm. (**B**) Size distribution of eMELNs analyzed by dynamic light scattering. (**C**) Zeta potential measurements of eMELNs, G-eMELNs, and TG-eMELNs. Data are presented as mean ± SD (*n* = 3). No significant differences in zeta potential were observed among groups (ordinary one-way ANOVA followed by Tukey’s post-hoc test, all *p* > 0.05; ns, not significant). (**D**) Western blot analysis of markers in MELNs p18, EAVs and eMELNs. (**E**) Confocal microscopy images of HaCaT cells incubated with DiI stained TAT-GFP-eMELNs and the Pearson’s correlation coefficient (r = 0.95455) confirms high co-localization between Dil and GFP signals, verifying successful fusion.

Therefore, we produced a series of eMELNs with different peptide segments from equal quality of MELNs p18 and HaCaT cell-derived EAVs containing different peptide segments (T stands for TAT, G stands for GFP, F stands for Flag). TEM and Malvern Particle Size Analyser images demonstrated that the lipid bilayer structure, size, and zeta potential of both eMELNs and G-eMELNs/TG-eMELNs were comparable (Figure 1A–C). These findings indicated that mixing of MELNs p18 with HaCaT EAVs and extruding again did not substantially alter the morphology or physicochemical properties of eMELNs from extruding MELNs p18. Western blot analysis indicated that flag was detected in F-eMELNs and TF-eMELNs, while GFP existed in G-eMELNs and TG-eMELNs (Figure 1D). Both TSG101 and CD63—the classical markers of mammalian EVs—were detectable in EAVs Lane. Neither TSG101 nor CD63 was detected in MELNs p18 Lane. And both TSG101 and CD63 reappeared as positive bands in e-MELNs Lane, though with weaker intensity compared to EAVs (Figure 1D). This evidence demonstrates that EAVs are indeed mammalian cell-derived (TSG101/CD63 positive); MELNs p18 are plant-derived (TSG101/CD63 negative); e-MELNs are true fusion products of both (TSG101/CD63 reappearance), rather than a simple physical mixture.

To observe whether the red and green fluorescence is co-localized, we stained MELNs p18 with DiI before mixing with TAT-GFP-HaCaT cell-derived EAVs to obtain DiI stained TAT-GFP-eMELNs and incubated HaCaT cells with DiI stained TAT-GFP-eMELNs (100 μg/mL) again. Confocal imaging and Pearson’s correlation coefficient (PCC) revealed that most of the red fluorescence colocalized with the green fluorescence (Figure 1E), which means most of the e-MELNs were loaded with TAT-GFP peptide successfully. We supplemented cryo-TEM micrographs of the mixed system before and after re-extrusion in Figure S1F. It can be observed that before extrusion, particles in p18 exhibited highly divergent sizes, leading to severe heterogeneity within the system. After extrusion, particle fragmentation and reassembly occurred, yielding a comparatively uniform particle population. Since the experimental strategy adopted here relies on fragmentation followed by reassembly, rather than direct vesicle fusion, the juxtaposition and fusion of two separate vesicles could not be readily visualized.

### 3.3. Permeation Evaluation of eMELNs with TAT Peptides

In order to observe the permeability of both eMELNs and drugs intuitively, we synthesized chebulinic acid with cy5.5 (Figure S2), electroporating cy-CA into eMELNs with or without TAT peptides. The method for electroporating drugs into eMELNs is as described in previous studies [6]. CCK-8 assays confirmed that 0.1–10 μg/mL CA/cy-CA and all 100 μg/mL eMELNs exhibited no significant cytotoxicity toward HaCaT cells (Figure S3A).

**Figure 2:**
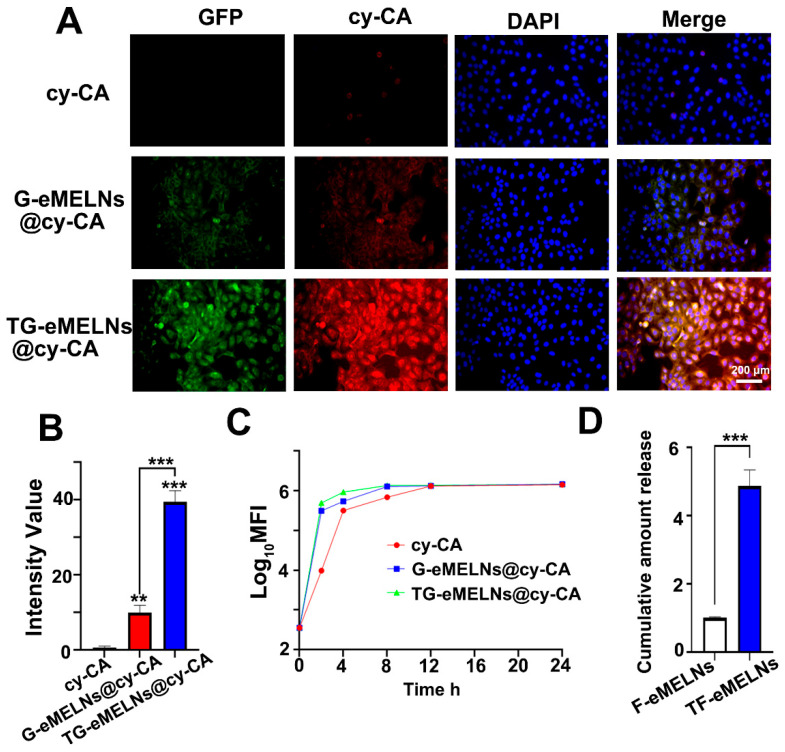
Permeation evaluation of eMELNs with TAT peptides in vitro. (**A**) Confocal microscopy images of HaCaT cells incubated with eMELNs@cy-CA. Scale bar: 200 μm. (**B**) Statistical results of intensity value in (**A**). Data are presented as mean ± SD (*n* = 3). Statistical significance: ** *p* < 0.01, *** *p* < 0.001. Ordinary one-way ANOVA followed by Tukey’s post hoc test. (**C**) Flow cytometry assay of HaCaT cells incubated with eMELNs@cy-CA to detect the changes in cy-CA absorption. (**D**) Franz diffusion cells were used for ex vivo permeation of F-eMELNs and TF-eMELNs. Mean difference = 3.83-fold, unpaired *t*-test, *p* = 0.0006; *** *p* < 0.001. Data are presented as mean ± SD (*n* = 3).

Then we incubated HaCaT cells with 10 μg/mL cy-CA, G-eMELNs@cy-CA and TG-eMELNs@cy-CA (containing 10 μg/mL cy-CA + 100 μg/mL eMELNs). After 30 min, it can be observed that the permeability of cy-CA loaded with eMELNs was significantly enhanced compared with directly incubating cy-CA, and the permeability of cy-CA loaded into eMELNs with TAT peptide was strongest (Figure 2A). The permeability of TG-eMELNs was also significantly enhanced compared to G-eMELNs (Figure 2A,B). To detect the changes in intracellular fluorescence intensity of cy-CA time-variation by flow cytometry, it was found that the TG-eMELNs@cy-CA group had the strongest fluorescence intensity within 8 h. After 12 h, the fluorescence levels of each group became close to each other due to the same total cy-CA concentration (Figure 2C). For F-eMELNs and TF-eMELNs, which did not have fluorescence themselves, the mouse skin penetration of TF-eMELNs was approximately 5 times that of F-eMELNs by FDC assay (Figure 2D).

**Figure 3:**
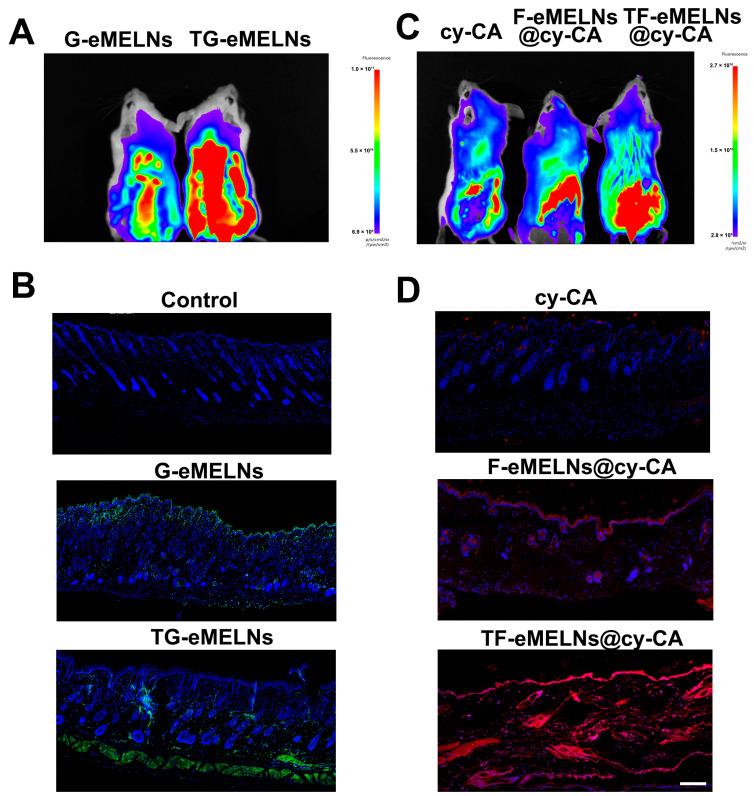
Permeation evaluation of eMELNs with TAT peptides in vivo. (**A**) In vivo imaging was used to test the penetration of G-eMELNs and TG-eMELNs. (**B**) Fluorescence sections after transdermal permeation in the G-eMELNs and TG-eMELNs. (**C**) In vivo imaging was used to test the penetration of cy-CA, F-eMELNs@cy-CA and TF-eMELNs@cy-CA. (**D**) Fluorescence sections after transdermal permeation in the cy-CA, F-eMELNs@cy-CA and TF-eMELNs@cy-CA. Scale bar: 200 μm.

G-eMELNs and TG-eMELNs were applied to the skins of shaved mice; a 7-day topical application study revealed that TAT peptide also significantly enhanced the skin permeability of eMELNs in vivo (Figure 3A,B). In similar experiments, the TAT peptide also enhanced the permeability of cy-CA loaded into TF-eMELNs compared with F-eMELNs@CA and free cy-CA (Figure 3C,D).

### 3.4. Functional Study of the CA and eMELNs@CA to Light Photoaging

Cold climate, hypobaric hypoxia, high altitude, robust UV radiation and prolonged sunshine hours constitute the key environmental characteristics of the Qinghai–Xizang Plateau [4]. To investigate the potential anti-UVA properties of natural Chinese medicine products, we selected *T. chebula*, renowned as the “King of Tibetan Medicine”, as a promising candidate for evaluating its anti-UVA efficacy.

**Figure 4:**
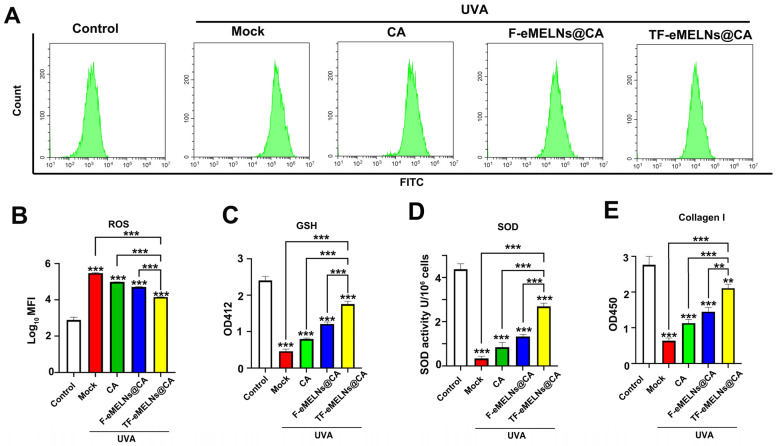
CA and eMELNs@CA restore ROS and collagan I levels. (**A**) Analysis of intracellular ROS levels in HaCaT cells after UVA and UVA + CA/eMELNs@CA treatments by flow cytometry. (**B**) Statistical results of (**A**). (**C**–**E**) Quantification of (**C**) intracellular glutathione (GSH) content, (**D**) superoxide dismutase (SOD) activity, and (**E**) collagen I levels after the indicated treatments. All data are presented as mean ± SD (*n* = 3). Statistical significance was determined using ordinary one-way ANOVA followed by Tukey’s post hoc test. ** *p* < 0.01, *** *p* < 0.001, as indicated.

**Figure 5:**
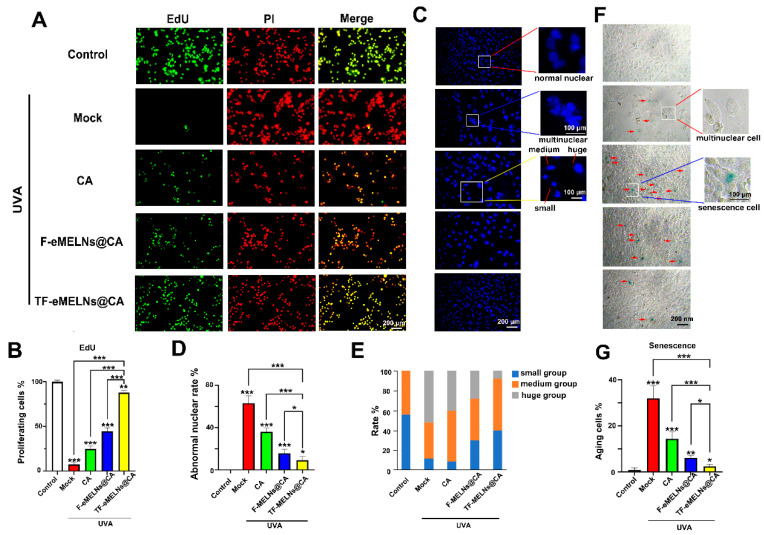
CA and eMELNs@CA alleviate UVA-induced cellular photoaging in HaCaT cells. (**A**) Representative images of EdU stained HaCaT cells after UVA and UVA + CA/eMELNs@CA treatments. Scale bar: 200 μm. (**B**) Statistical results of (**A**). (**C**) DAPI staining was used for analysis of the multinuclear/micronuclei and nuclear size in UVA and UVA + CA/eMELNs@CA-treated HaCaT cells. The cell nuclei were classified as small, medium and large groups. (**D**,**E**) Statistical results of (**C**). (**F**) Representative images of β-galactosidase-stained HaCaT cells after UVA and UVA + CA/eMELNs@CA treatments. The red arrow indicates senescent cells. (**G**) Statistical results of (**F**). All quantitative data are presented as mean ± SD (*n* = 3). Statistical significance was determined using ordinary one-way ANOVA followed by Tukey’s post hoc test. * *p* < 0.05, ** *p* < 0.01, *** *p* < 0.001, as indicated.

UVA irradiation promotes the accumulation of ROS and subsequently leads to cell senescence. To evaluate the ROS levels, we utilized an ROS assay kit combined with flow cytometry. The results demonstrated that UVA treatment significantly increased ROS levels, which were restored by CA (Figure 4A,B). Collagen type I is the most abundant collagen in skin and is produced by fibroblasts [25]. Glutathione (GSH) and superoxide dismutase (SOD) function as central antioxidants [26], so GSH and SOD detection kits were applied to analyze the effect of CA on the increase in ROS caused by UVA. GSH and SOD levels in the UVA group decreased significantly, while it improved after CA treatment (Figure 4C,D). The collagen I level in HFF cells was restrained after UVA treatment and restored by CA (Figure 4E). The 5-ethynyl-2’-deoxyuridine (EdU) cell proliferation assay revealed that the proportion of proliferating cells decreased following UVA irradiation but was restored by CA (Figure 5A,B). We observed an increase in multinuclear/micronuclei and nuclear size in HaCaT cells exposed to UVA, which are hallmarks of DNA damage and cellular aging [25,26], while CA and e-MELNs@CA alleviated these phenomena (Figure 5C–E). Furthermore, β-Galactosidase staining confirmed that UVA irradiation induced cell senescence, which was alleviated by CA (Figure 5F,G). And F-eMELNs@CA exhibited stronger resistance to the growth inhibition, aging, ROS activation, collagen I, GSH and SOD decrease induced by UVA, while TF-eMELNs@CA exhibited the strongest resistance (Figure 4 and Figure 5).

Approximately 55% of CA was encapsulated into eMELNs, leaving 45% free CA. We treated UVA-irradiated HaCaT cells with three groups of samples: a CA solution containing CA at an equivalent concentration to free CA, blank TAT-eMELNs without CA loading, and CA-loaded eMELNs with free CA removed. Afterwards, we measured cellular apoptosis and the elevated ROS levels (Figure S4). The results revealed that the blank TAT-eMELN group exhibited nearly no restorative effect; the CA solution with matching free CA concentration only exerted a slight protective effect; by contrast, CA-loaded eMELNs depleted of free CA retained prominent restorative capacity. These findings demonstrate that the therapeutic restoration effect of our drug-loaded system primarily stems from CA delivered and permeabilized by vesicles, with only a minor contribution from free CA, while the carrier itself possesses negligible therapeutic activity.

### 3.5. CA and eMELNs@CA Combat Skin Light Photoaging Caused by UVA

**Figure 6:**
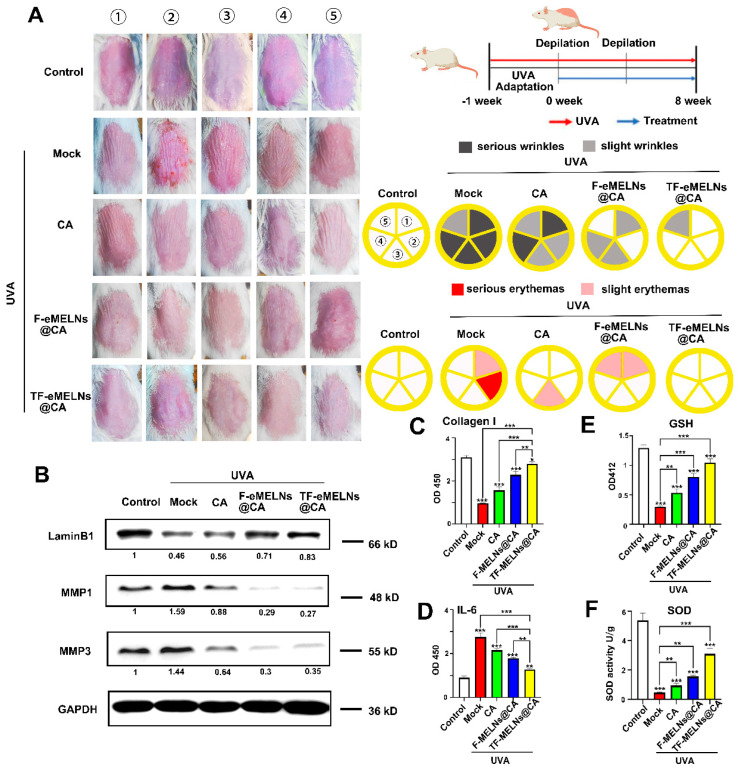
CA and eMELNs@CA combat skin light photoaging caused by UVA. (**A**) Representative images of mouse dorsal skin after 8 weeks of different topical treatments. (**B**) Western blot analysis of LaminB1 and MMPs proteins in skins after 8 weeks of different topical treatments. Relative protein expression levels are indicated below each band, normalized to the control group. (**C**–**F**) Quantification of (**C**) collagen I, (**D**) IL-6, (**E**) GSH, and (**F**) SOD activity in mouse skin tissues after 8 weeks of treatment. All quantitative data are presented as mean ± SD (*n* = 5). Statistical significance was determined using ordinary one-way ANOVA followed by Tukey’s post hoc test. * *p* < 0.05, ** *p* < 0.01, *** *p* < 0.001, as indicated.

We established a murine model of UVA-induced skin damage to investigate the in vivo effects of CA and eMELNs@CA. After 8 weeks of treatments, TUNEL staining confirmed the absence of significant skin damage following the topical application of eMELNs@CA (Figure S3B). And the effects of eMELNs@CA on the major organs of the mice were similar to those of PBS (Figure S3C). And the control group presented a smooth skin surface with no significant damage, whereas the UVA-exposed mock group presented evident wrinkles and erythema. Different treatments alleviated skin tissue damage to varying degrees, with TF-eMELNs@CA demonstrating the most marked effect (Figure 6A). To quantitatively evaluate the alleviation of UVA-induced skin photoaging phenotypes by different formulations, we established a standardized 0–3 semi-quantitative scoring system to separately assess wrinkle severity and erythema severity on the dorsal skin of mice. All photographs of mouse back skin were randomly reordered and blindly scored by two independent researchers to eliminate subjective bias. The scoring criteria were defined as follows. For wrinkle evaluation: 0 points represented completely smooth skin without visible folds (consistent with the untreated Control group); 1 point indicated faint superficial fine lines with no obvious skin depression; 2 points corresponded to distinct deep folds accompanied by rough skin texture; 3 points referred to extensive thick, deep wrinkles with evident skin thickening and collapse. For erythema evaluation: 0 points denoted uniform pale pink skin without any erythema; 1 point represented local faint erythema without edema or papules; 2 points indicated widespread obvious erythema and mild skin swelling; 3 points stood for severe deep red erythema accompanied by visible papules and tiny skin lesions. Each experimental group contained five individual mice, and wrinkle and erythema scores were assigned to each mouse according to the above criteria. The average score ± standard deviation of wrinkle and erythema for each group was calculated and is summarized in Table 1.

A significant loss of LaminB1, a major component of the nucleus, is a hallmark of senescent cells [27]. Collagen fibers primarily consist of collagen, and MMPs are the main proteases responsible for collagen breakdown [28]. CA and eMELNs@CA treatment effectively inhibited the decrease in LaminB1 and the expression of MMP-1 and MMP-3 in skins exposed to UVA (Figure 6B). Moreover, the erythema caused by a skin injury implied inflammation; CA and eMELNs@CA reduced the secretion of inflammatory factor IL-6 after UVA exposure (Figure 6C), while collagen I, GSH and SOD levels in the UVA group decreased significantly, and it improved after CA and eMELNs@CA treatment (Figure 6D–F).

**Figure 7:**
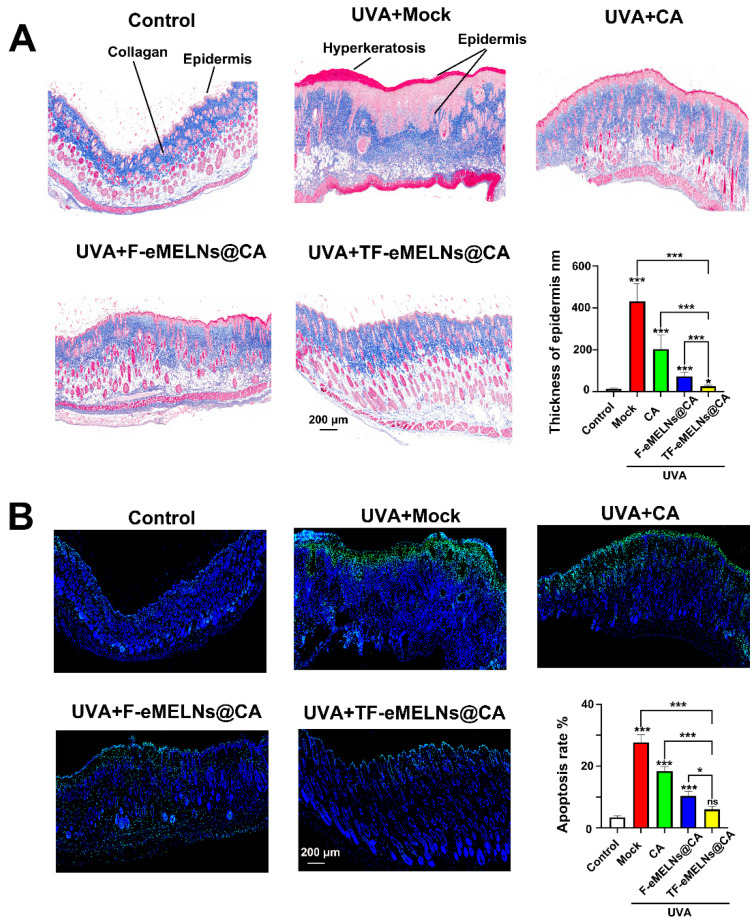
CA and eMELNs@CA alleviate UVA-induced structural damage and apoptosis in mouse skin. (**A**) Representative images of Masson staining of the dorsal skin of mice subjected to different 8-week topical treatments. Scale bar: 200 μm. (**B**) Representative images of TUNEL staining of the dorsal skin of mice subjected to different 8-week topical treatments. Scale bar: 200 μm. All quantitative data are presented as mean ± SD (*n* = 5). Statistical significance was determined using ordinary one-way ANOVA followed by Tukey’s post hoc test. * *p* < 0.05, *** *p* < 0.001, as indicated, ns, not significant.

Masson staining revealed that the control group had clear and intact collagen fibers with a thin and uniform epidermis. In contrast, the model group exhibited broken and uneven collagen fibers with hyperkeratosis of the stratum corneum and irregular thickening of the epidermis, indicative of photoaged skin (Figure 7A). Consistently, the rate of apoptotic cells increased markedly in the UVA model group (Figure 7B), whereas CA and eMELNs@CA treatments mitigated hyperkeratosis, irregular thickening of the epidermis and apoptosis, and the TF-eMELNs@CA demonstrating the most marked effect (Figure 7).

**Figure 8:**
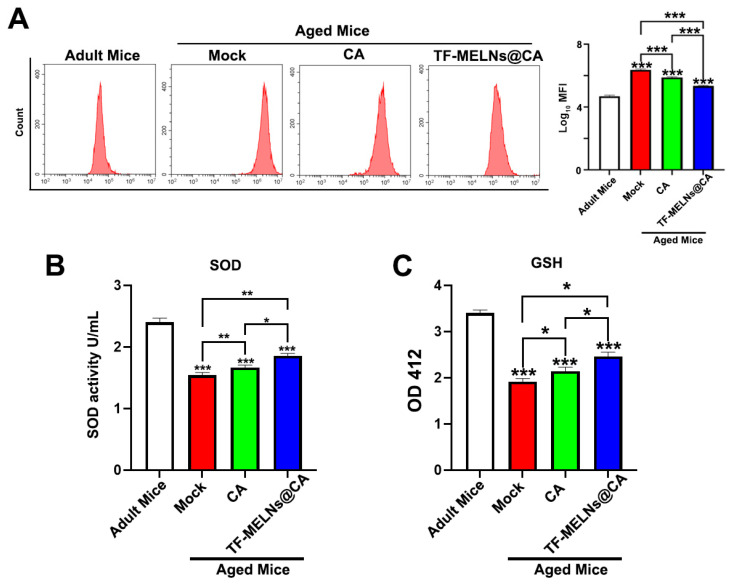
CA and eMELNs@CA has a therapeutic effect on naturally aging mice. (**A**) Analysis of intracellular ROS levels in skins after 8 weeks of different topical treatments by flow cytometry. (**B**,**C**) Quantification of (**B**) superoxide dismutase (SOD) activity and (**C**) glutathione (GSH) content in the skin of adult and aged mice after 8 weeks of treatment. All quantitative data are presented as mean ± SD (*n* = 5). Statistical significance was determined using ordinary one-way ANOVA followed by Tukey’s post hoc test. * *p* < 0.05, ** *p* < 0.01, *** *p* < 0.001, as indicated.

Additionally, after 8 weeks of treatments, detecting the levels of ROS, GSH and SOD in the blood of aged mice (18 months) and adult mice (2 months) revealed that CA and eMELNs@CA also has a therapeutic effect on naturally aging mice (Figure 8).

### 3.6. Mechanisms of Anti-Photoaging Aging

**Figure 9:**
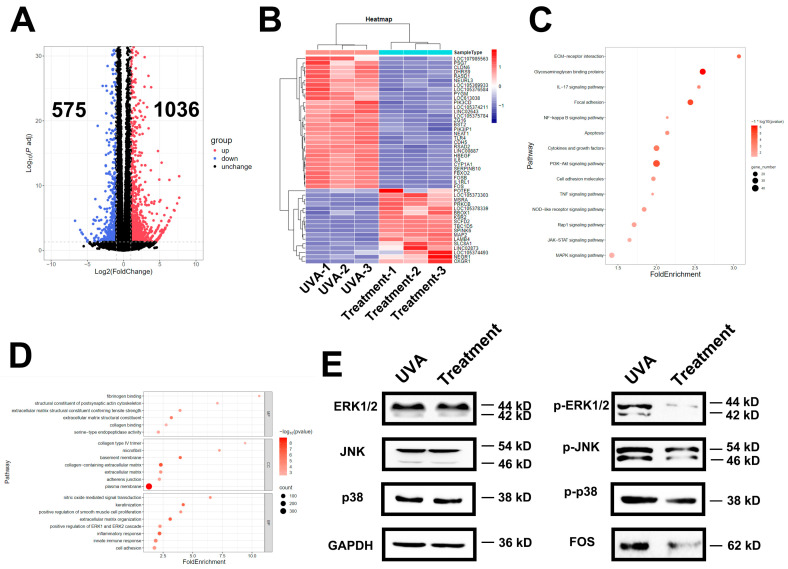
CA has an anti-UVA effect by altering the expression levels of MAPK signaling pathway components. (**A**–**D**) Gene ontology enrichment analysis of DEGs and enrichment analysis of the KEGG pathway. (**E**) Western blot analysis of MAPK family and FOS proteins in HaCaT cells after UVA and UVA + TF-eMELNs@CA treatments.

To further clarify the molecular mechanism underlying the anti-photoaging capacity of our delivery system, transcriptome sequencing was conducted on UVA-damaged HaCaT cells with and without receiving TF-MELNs@CA intervention. Differential gene expression analysis identified a total of 1611 differentially expressed genes (DEGs) between the two groups, consisting of 1036 upregulated genes and 575 downregulated genes (Figure 9A). Compared with the model group, the expression levels of FOS, FOSB and IL6 were downregulated in the treatment group, suggesting that the treatment might inhibit UVA-induced inflammatory activation (Figure 9B). The extracellular matrix (ECM) governs skin elasticity and tensile resilience. A structurally intact ECM serves as an essential prerequisite to support the normal adhesion, spreading, migration and physiological function of fibroblasts. KEGG pathway enrichment analysis of DEGs showed IL-17, NF-κB, PI3K, TNF, Rap1, JAK and MAPK pathways were involved (Figure 9C). Gene ontology function enrichment analysis of DEGs revealed that our treatment significantly enriched key pathways of ECM, including extracellular matrix structural constituent, collagen-containing extracellular matrix, and extracellular matrix organization. Enrichment of collagen binding, collagen type IV trimer, and basement membrane terms further indicates direct regulation of ECM synthesis, assembly, and structural stability (Figure 9D). Concomitant enrichment of inflammatory and innate immune response pathways suggests the treatment also creates a favorable microenvironment for repair. Notably, ERK1/2, a member of the MAPK family, was significantly enriched in the Biological Process (BP) category. To further validate the involvement of the MAPK signaling pathway in the anti-photoaging effect of the treatment group, we performed Western blot analysis to examine the expression and phosphorylation status of MAPK pathway components. As shown in Figure 9E, compared with the UVA model group, the treatment group exhibited a notable reduction in the phosphorylation levels of ERK1/2, JNK, and p38 kinases, while the total protein levels of these kinases remained unchanged. Consistently, the expression of FOS, a downstream transcriptional effector of the MAPK pathway, was significantly downregulated in the treatment group relative to the UVA group. These findings were consistent with our KEGG/GO pathway enrichment results and transcriptome heatmap data.

Together, these results confirmed that our treatment inhibits UVA-induced activation of the MAPK signaling cascade, thereby suppressing the downstream expression of pro-inflammatory transcription factors such as FOS, promoting ECM repair and tissue remodeling by enhancing collagen production, stabilizing ECM structure, and resolving inflammation, which contributed to the attenuation of skin photoaging.

## 4. Discussion

Our previous work has confirmed that CA, a core bioactive component extracted from Terminalia chebula (known as “King of Tibetan Medicine”), demonstrates ROS-scavenging capacity and efficiently repairs UVB-induced cutaneous photodamage both in vitro and in vivo [6]. Building on this foundation, the present study further expands the anti-photoprotective spectrum of CA and systematically demonstrates its robust therapeutic efficacy against UVA-induced skin photoaging, which is predominantly responsible for long-term cutaneous oxidative stress, ECM degradation and irreversible skin senescence. More importantly, we successfully constructed a novel membrane hybridization engineering strategy to fabricate eMELNs with cell-penetrating peptide (TAT) by fusing waste-derived large Mirabilis himalaica particle sediments (MELNs p18) with HaCaT cell-derived extracellular artificial vesicles (EAVs).

In classical vesicle–vesicle fusion studies, “fusion” typically describes two intact, separate liposomes slowly approaching, docking, and merging under mild conditions—a process that takes seconds to minutes and is readily visualized by electron microscopy. In contrast, the extrusion-based system reported here proceeds through a fundamentally different mechanism. When both cell membranes and liposomal membranes are forcibly passed through a nanopore, they undergo severe mechanical fragmentation. Within milliseconds upon exiting the pore, these metastable membrane fragments rapidly reassemble to minimize edge tension, resulting in the mixing of bilayer components. Thus, fusion in this context does not occur between two pre-existing intact vesicles, but rather between membrane nanodisks during instantaneous recombination.

Given the sub-millisecond timescale within the nanochannel, direct visualization of the classical fusion intermediate (i.e., two juxtaposed and merging vesicles) is technically unfeasible. However, the absence of such snapshots does not negate the occurrence of membrane fusion; it merely indicates that our approach follows a non-classical, shear-driven pathway. The final hybrid membrane is generated through bilayer breakage and reconnection, not through lipid exchange or physical adsorption—a distinction that we explicitly emphasize in defining our method.

This interpretation is consistent with recent studies that employ nearly identical extrusion strategies to generate hybrid vesicles from plant and animal sources [29,30]. Notably, those reports also rely on fluorescence colocalization, protein co-existence (Western blot), and functional enhancement as evidence for fusion, without providing TEM images of fusion intermediates or FRET-based lipid mixing assays. Given the technical constraints of capturing millisecond events within nanoscale channels, the field currently accepts colocalization combined with functional gain as reasonable evidence for extrusion-induced membrane hybridization. Our findings are therefore validated using the same established criteria.

In order to resolve the innate shortcomings of PELNs, scientists have devised numerous engineering modification approaches to synthesize high-efficiency modified plant-derived nanovesicles, which facilitate deep dermal permeation, accurate lesion targeting, and effective drug transport. Chemical modification modifies vesicle surfaces via covalent and non-covalent bonding to elevate cellular uptake rates, drug loading capacity and biological safety. By physical or chemical means, membrane hybridization merges PELN membranes with mammalian or bacterial cell membranes, creating hybrid delivery systems with better immune evasion as well as enhanced targeting effects toward inflammatory and tumor sites [31]. And our green, agent-free membrane fusion platform effectively transforms underutilized plant-derived particulate waste into high-efficiency, biocompatible transdermal nanocarriers, achieving significantly enhanced intracellular and cutaneous delivery of CA and thereby superior anti-UVA photoaging effects compared with free CA and non-TAT-modified nanocarriers.

Consistent with previous natural product and nanocarrier photoprotection studies, persistent UVA irradiation triggers excessive intracellular ROS accumulation, which disrupts endogenous antioxidant defense systems characterized by decreased SOD activity and GSH depletion, ultimately leading to oxidative stress-mediated cell cycle arrest, nuclear morphological abnormality, and cellular senescence [32,33]. In agreement with these classical photoaging mechanisms, our in vitro results verified that UVA exposure dramatically elevated ROS levels, suppressed antioxidant enzyme activity, reduced collagen I synthesis, impaired HaCaT cell proliferation, and induced nuclear damage and senescence. Notably, both free CA and eMELNs@CA treatments significantly reversed UVA-induced oxidative injury and senescent phenotypes, whereas TAT-modified TG-eMELNs@CA exhibited the most prominent protective effect. This superior performance can be fully explained by our permeation validation results: compared with 293T cell-derived EAVs, HaCaT-derived EAVs possessed inherently stronger transdermal penetration ability, and further TAT peptide modification significantly improved the transdermal and intracellular delivery efficiency of nanocarriers. These findings align with extensive literature confirming that TAT cell-penetrating peptides substantially enhance the transmembrane and cutaneous accumulation of nanomedicines, overcoming the stratum corneum barrier limitation that severely restricts the clinical and topical application of most natural small-molecule drugs.

Mechanistically, transcriptomic sequencing was performed to systematically explore the underlying molecular regulatory network of TG-eMELNs@CA against UVA photoaging. A total of 1611 differentially expressed genes were identified between the UVA model and TG-eMELNs@CA treatment groups, providing comprehensive transcript-level evidence for the anti-photoaging function of our nanosystem. KEGG enrichment analysis revealed the significant involvement of classic photoaging-related pathways, including MAPK, NF-κB, PI3K, TNF, and IL-17 signaling pathways [7]. Among these, the MAPK signaling cascade, which serves as the core hub linking oxidative stress, inflammation, and ECM remodeling during skin photoaging, was prominently enriched. Consistent with transcriptomic data, Western blot validation confirmed that UVA irradiation markedly induced the phosphorylation of ERK1/2, JNK, and p38, thereby activating downstream pro-senescence and pro-inflammatory transcription factors such as FOS and FOSB [34]. In contrast, TG-eMELNs@CA treatment effectively suppressed the phosphorylation of MAPK family kinases and inhibited the overexpression of FOS and IL6, thereby blocking UVA-triggered inflammatory activation. GO functional enrichment further demonstrated that our treatment significantly restored ECM structural stability by regulating collagen-containing extracellular matrix organization, collagen binding, and basement membrane assembly, which effectively explained the recovered collagen I content and repaired dermal tissue structure in both in vitro and in vivo models. Collectively, these multi-level results illustrate that the TAT-functionalized eMELNs@CA nanosystem alleviates UVA-induced skin photoaging via inhibiting MAPK-mediated oxidative inflammation and promoting ECM structural repair, which complements and expands the existing mechanistic understanding of natural product-based photoprotective agents.

In vivo UVA-induced photoaging mouse models further validated the translational potential of eMELNs@CA. Long-term UVA exposure caused obvious skin wrinkling, erythema, epidermal hyperkeratosis, dermal collagen fiber breakage, and excessive cutaneous cell apoptosis, accompanied by decreased Lamin B1 expression, elevated MMP1/MMP3 collagen-degrading enzyme levels, and increased IL-6 inflammatory secretion. In accordance with in vitro trends, topical administration of eMELNs@CA significantly improved macroscopic skin appearance, alleviated epidermal hyperplasia and dermal structural damage, reduced apoptotic cell numbers, restored antioxidant levels, and inhibited inflammatory responses and collagen degradation. Moreover, the natural aging mouse model further confirmed that CA-loaded TAT-modified nanocarriers could effectively ameliorate age-related oxidative decline in skin tissue, demonstrating that this nanoplatform exerts protective effects against both acute UVA-induced photoaging and chronic natural skin aging. Importantly, safety evaluation verified that eMELNs exhibited negligible cytotoxicity in HaCaT cells within the working concentration range and caused no obvious pathological damage to major organs after long-term topical application, confirming excellent biocompatibility and topical safety, which is essential for future dermatological application.

Notably, the innovation of this study lies not only in the expanded anti-photoaging efficacy of CA but also in the optimized resource utilization and biosafety of the nanocarrier preparation strategy. Most plant-derived exosome-like nanoparticle studies only utilize high-purity small-size nanoparticles and discard large-particle sediments generated during centrifugation, resulting in severe resource waste. In this work, we innovatively repurposed the discarded large-size MELNs p18 sediments as raw carrier materials and fabricated high-performance transdermal nanocarriers through simple physical extrusion and membrane hybridization with HaCaT-derived EAVs. This preparation process avoids chemical modification and toxic crosslinking agents, ensures high biological safety, and greatly improves the utilization rate of Mirabilis himalaica biological resources, providing a new resource-efficient preparation paradigm for natural product nanocarrier engineering. Meanwhile, the membrane-anchored TAT peptide successfully endows the plant-derived nanocarrier with superior transdermal penetration capability, solving the bottleneck of poor skin permeability and low bioavailability of traditional plant active ingredients.

Despite the promising findings of the present study, several limitations still need to be acknowledged and addressed in future investigations. First, although we confirmed that TAT peptide modification enhances transdermal and intracellular delivery efficiency of eMELNs, the exact membrane fusion mechanism between MELNs and HaCaT EAVs, as well as the precise molecular interaction between TAT peptides and skin stratum corneum lipids, requires further in-depth exploration through molecular docking and membrane permeability mechanism experiments. Second, our transcriptomic analysis focused on the MAPK signaling pathway; however, other enriched pathways (e.g., NF-κB and PI3K) may also participate in the anti-photoaging effect of eMELNs@CA, and their potential synergistic regulatory mechanisms were not fully validated in this study. Third, all in vivo experiments were performed on mouse skin models, and differences in skin structure and permeability between mice and humans may limit the direct translational application of this nanosystem; future human or pig skin [35] explant experiments and clinical trials are required to verify clinical efficacy. Fourth, this study only explored the short-term topical therapeutic effect of eMELNs@CA, and the long-term skin tolerance, drug retention, and optimal administration cycle still need systematic optimization.

## 5. Conclusions

Our previous study demonstrated that the anti-ROS effect of T. chebula extract is caused mainly by CA and that CA effectively repaired UVB-induced skin damage both in vivo and in vitro [6]. In this study, we further proved that CA was an effective ingredient of resistance to UVA-induced photoaging. We obtained TAT-eMELNs by mixing HaCaT cell-derived EAVs with MELNs p18 and extruding them through a filter membrane for membrane hybridization. This strategy first circumvents the need for supplementary chemical agents, thereby alleviating concerns over potential biosafety hazards. Second, it converts non-PELN macro-particles into high-value carriers, which serves to elevate the utilization efficiency of biological resources. Finally, the anchoring of TAT peptides onto the membrane surface markedly enhances the cellular and cutaneous uptake of EAVs. In addition, we encapsulated CA into TAT-modified eMELNs (TAT-eMLNs@CA) via electroporation, and subsequent results verified the anti-UVA activity of both free CA and TAT-eMLNs@CA through in vitro and in vivo assays. Compared with free CA or CA in eMELNs without TAT peptides, TAT-eMELNs@CA exhibited the strongest permeation into cells and skins, which resulted in the most significant protection against UVA damage. Based on prior research findings, this work further broadens the translational prospects of CA and PELNs for applications in dermal therapeutics and ultraviolet radiation protection.

## Figures and Tables

**Table 1:** Semi-quantitative scoring results of wrinkle and erythema severity for all experimental groups.

Group	Mean Wrinkle Score ± SD	Mean Erythema Score ± SD
Control	0.00 ± 0.00	0.00 ± 0.00
Mock	2.20 ± 0.75	1.60 ± 0.80
CA	1.20 ± 0.40	0.60 ± 0.49
F-eMELNs@CA	0.80 ± 0.40	0.40 ± 0.49
TF-eMELNs@CA	0.40 ± 0.49	0.20 ± 0.40

Note: The blind semi-quantitative scoring results of wrinkle and erythema severity based on a standardized 0–3 scoring scale of Figure 6A.

## Data Availability

The data presented in this study are available on request from the corresponding author due to privacy restrictions.

## Supplementary Materials

The following supporting information can be downloaded at: https://www.mdpi.com/article/10.3390/cells15141235/s1, Figure S1: Screening of Preparation condition. Figure S2: The structure and verification of cy-CA. Figure S3: Safety of CA, cy-CA and eMELNs@CA. Figure S4: Therapeutic efficacy evaluation of free CA and blank carriers.
