# Supplementary material for: Membrane Fusion-Based Mirabilis Himalaica-Derived Exosome-like Nanoparticles Fused with Cell-Penetrating Peptide Mediated for Chebulinic Acid Delivery Against UVA-Induced Photoaging
Source: Cells. 2026 Jul 8;15(14):1235. doi: 10.3390/cells15141235 (PMC13406704; doi:10.3390/cells15141235)
Supplement: Supplementary file 1 [file cells-15-01235-s001.zip › cells-4361751-supplementary.pdf]

## Supplementary Figures

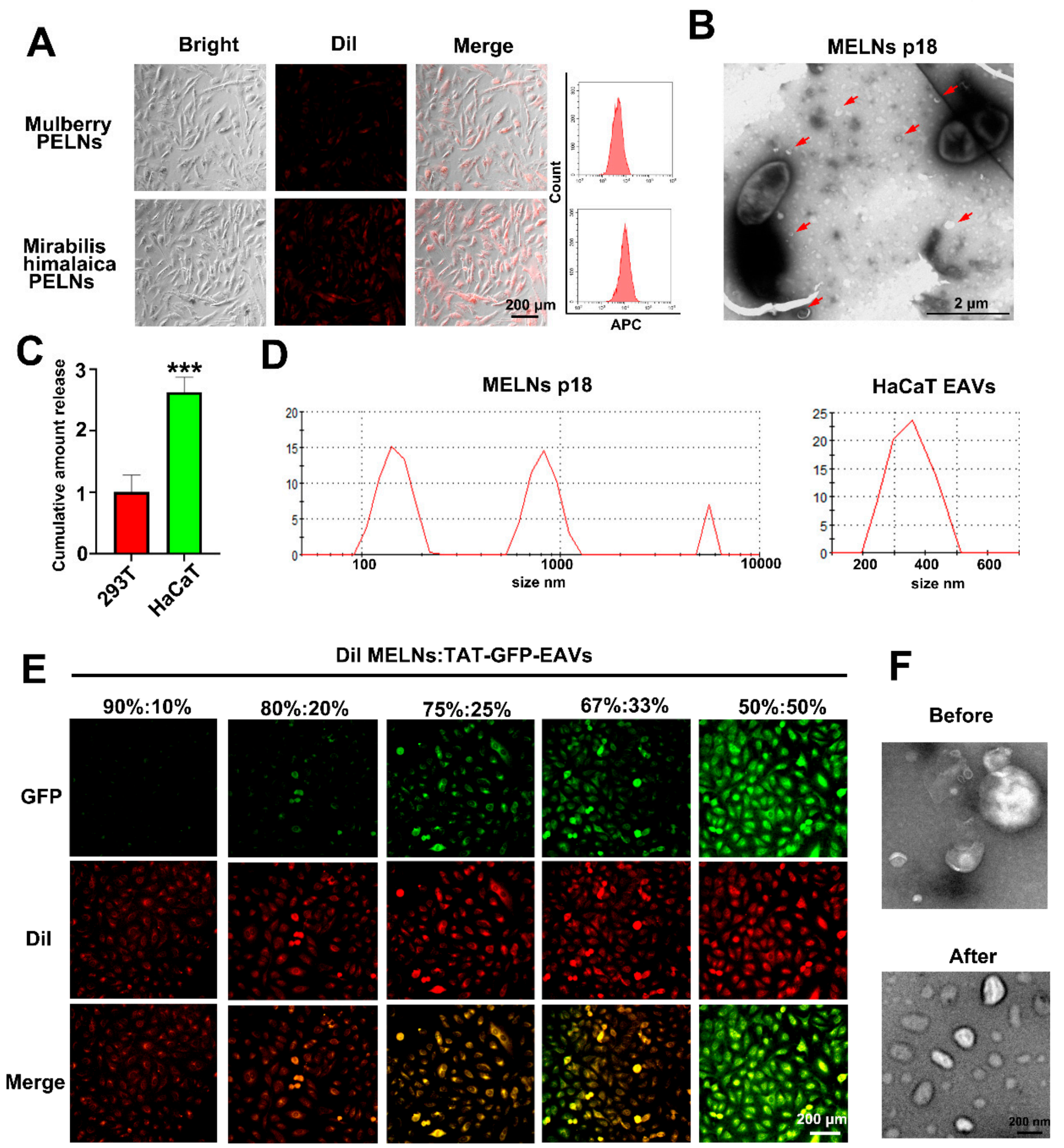


**Figure S1 Screening of Preparation condition.** (A) Representative images of the DiI-positive HFF cells at the bottom of the plates when DiI stained nanoparticles were added to the upper chamber. Scale bar:200 μm. The flow cytometry results of the bottom HFF cells were consistent with it. (B) The morphology of MELNs p18 characterized using TEM, MELNs were marked by red arrows. Scale bar: 2 μm. (C) Ex vivo skin permeation of 293T cell-derived extracellular vesicles (EAVs) and HaCaT cell-derived EAVs, measured using Franz diffusion cells. The cumulative amount released was significantly higher for HaCaT EAVs than for 293T EAVs (mean difference=1.68-fold, unpaired t-test, $p=0.0007$; ***$p < 0.001$). Data are presented as mean±SD ($n$=3). (D) Size distribution analyses of MELNs p18 and HaCaT EAVs. (E) Confocal microscopy images of HaCaT cells incubated with DiI stained TAT-GFP-eMELNs from different proportions of raw materials. Scale bar:200 μm. (F) The morphology of mixed system before and after extrusion characterized using TEM. Scale bar: 200 nm.

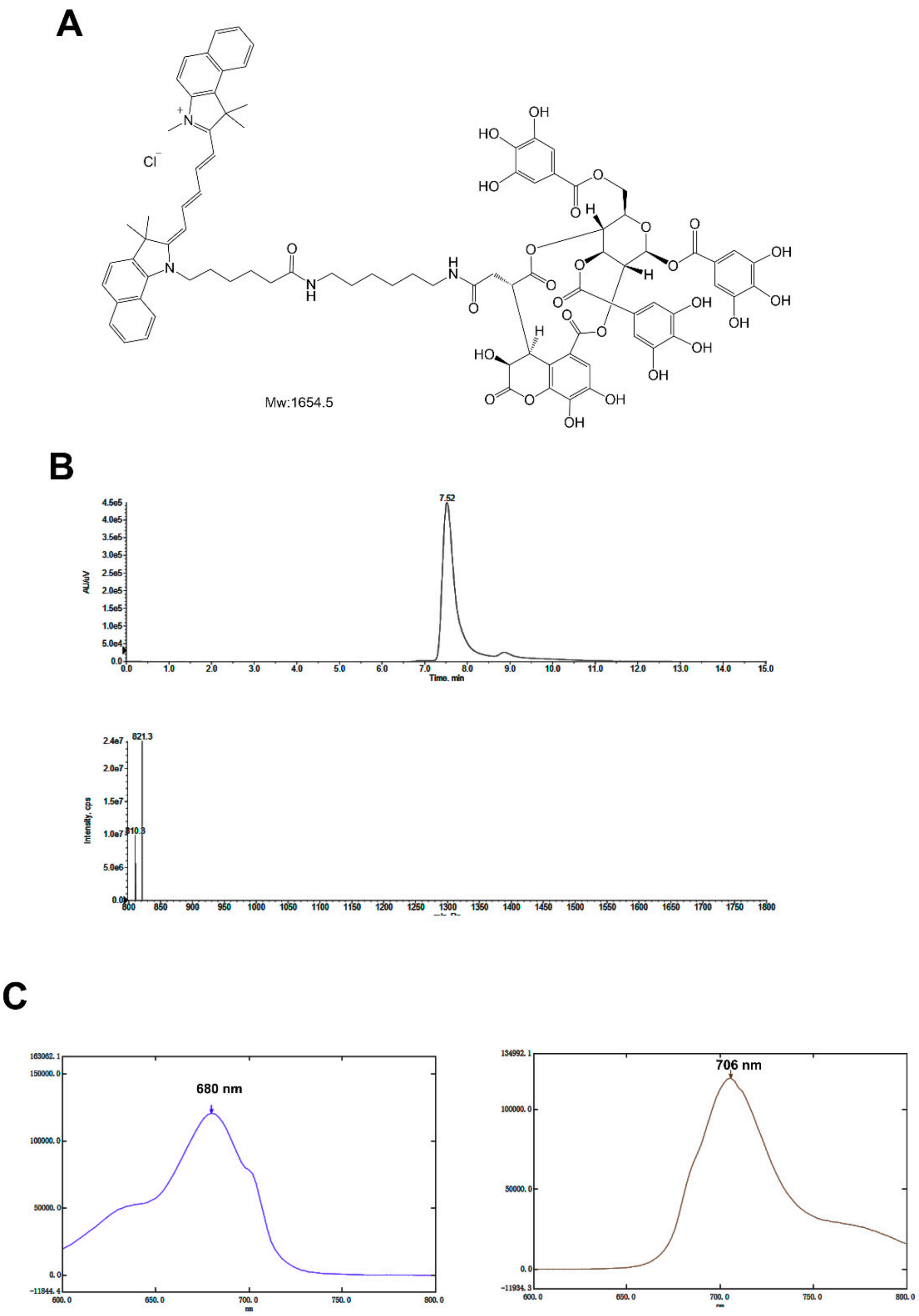


**Figure S2 The structure and verification of cy-CA.** (A) The structure of cy-CA, Mw=1654,5. (B) Mass spectrum of cy-CA, Mw of cy-CA without $Cl^-$ =1619.6, (M+H)/2=810.3, (M+Na)/2=821.3. (C) The wavelength of maximum absorption and emission of cy-CA.

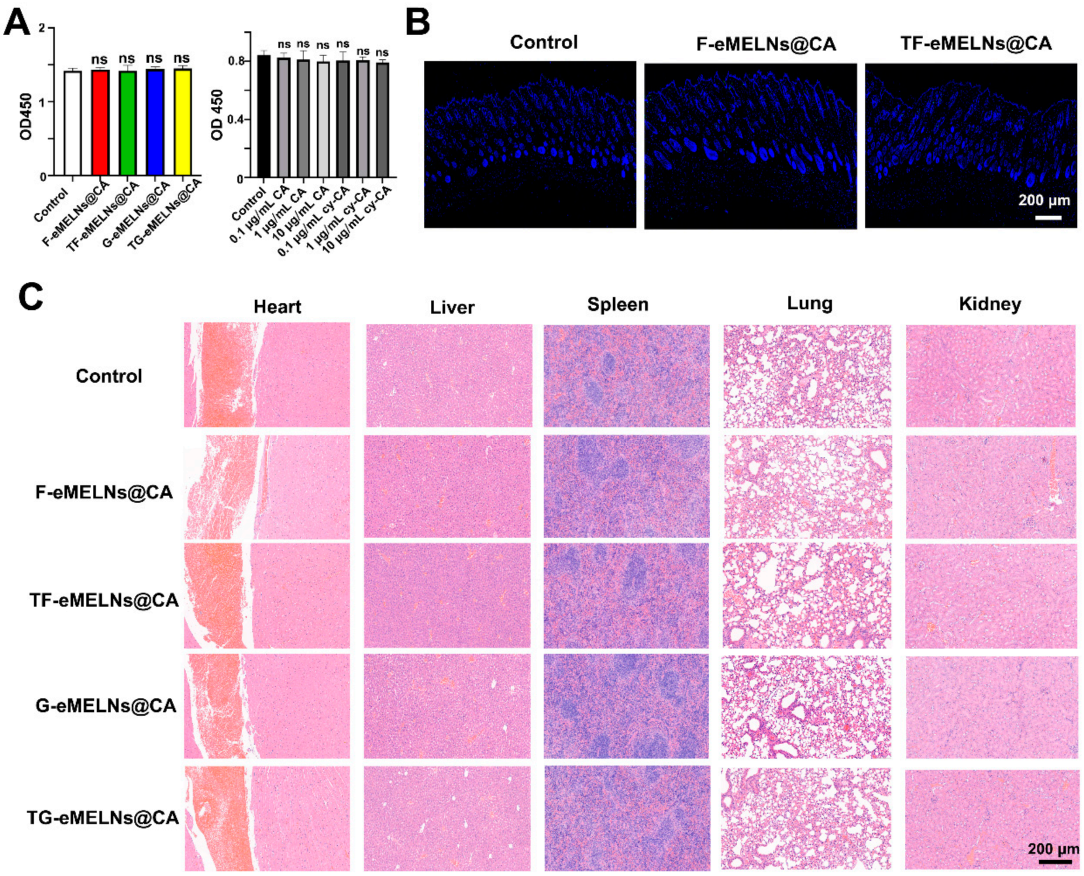


**Figure S3 Safety of CA, cy-CA and eMELNs@CA.** (A) *In vitro* cytotoxicity assay of HaCaT cells after treatment with CA, cy-CA, and eMELNs@CA formulations (CCK-8 assay). Data are presented as mean±SD ($n$=3). No significant differences vs. control were observed (ordinary one-way ANOVA followed by Tukey's post-hoc test, all p>0.05; ns, not significant). (B) Representative images of Tunel stained skins after 8 weeks topical application of F-MELNs@CA and TF-MELNs@CA. Scale bar:200 µm. (C) H&E staining of major organs in mice after 8 weeks topical application of eMELNs. Scale bar:200 µm.

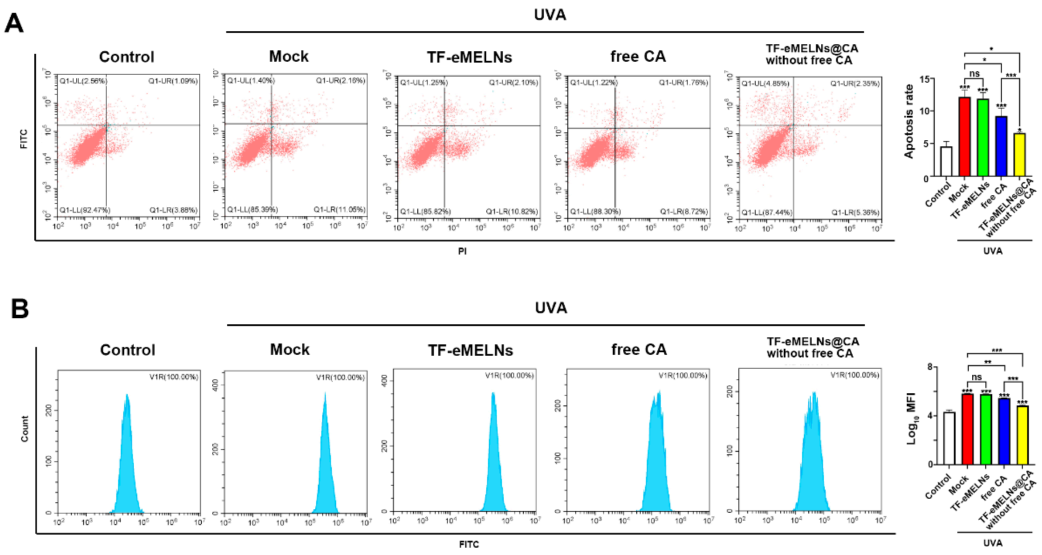

**Figure S4 Therapeutic efficacy evaluation of free CA and blank carriers.** Analysis of apoptosis rate (A) and intracellular ROS levels (B) in HaCaT cells after different treatments by flow cytometry. All quantitative data are presented as mean±SD (*n*=3). Statistical significance was determined using ordinary one-way ANOVA followed by Tukey's post-hoc test. *$p<0.05$, **$p<0.01$, ***$p<0.001$, as indicated.
